# Ultrasonographic Diagnosis of an Ischiocondylar Adductor Magnus Partial Tear: An Uncommon Cause of Ischial Pain

**DOI:** 10.3390/diagnostics16162583

**Published:** 2026-08-15

**Authors:** Chia-Yen Hsu, Wei-Ting Wu, Yu-Chun Hsu, Ke-Vin Chang, Levent Özçakar

**Affiliations:** 1 Department of Physical Medicine and Rehabilitation, National Taiwan University Hospital, Taipei 108207, Taiwan; graceht2@gmail.com; 2 Department of Physical Medicine and Rehabilitation and Community and Geriatric Research Center, National Taiwan University Hospital, Bei-Hu Branch, Taipei 108206, Taiwan; viph062@gmail.com; 3 Department of Physical Medicine and Rehabilitation, National Taiwan University College of Medicine, Taipei 100233, Taiwan; 4 Center for Regional Anesthesia and Pain Medicine, Wang-Fang Hospital, Taipei Medical University, Taipei 110301, Taiwan; 5 Department of Physical and Rehabilitation Medicine, Hacettepe University Medical School, Ankara 06100, Turkey; lozcakar@gmail.com

**Keywords:** hip pain, adduction, tendon, ultrasound, rehabilitation

## Abstract

Pain arising from the ischial region is commonly attributed to proximal hamstring injury or ischial bursitis, whereas injury to the ischiocondylar portion of the adductor magnus remains less commonly considered because of its deep location and complex anatomy. We report a 63-year-old woman who developed persistent proximal posterior thigh pain after sprinting, followed by progressive sitting pain despite conservative treatment. High-resolution musculoskeletal ultrasonography revealed focal structural abnormalities of the ischiocondylar portion of the adductor magnus at the tendon insertion and musculotendinous junction that were consistent with a partial tear, accompanied by surrounding inflammatory changes and ischial bursal fluid. The lesion was identified beginning with the short-axis view of the ischial tuberosity, followed by an oblique long-axis view of the ischiocondylar tendon. Conservative rehabilitation, activity modification, and low-level laser therapy resulted in complete symptom resolution without recurrence. This report emphasizes that injury of the ischiocondylar portion of the adductor magnus represents an uncommon differential consideration in patients presenting with proximal posterior thigh or ischial pain. A systematic ultrasound examination with appropriate transducer orientation facilitates accurate diagnosis and appropriate management.

**Figure 1:**
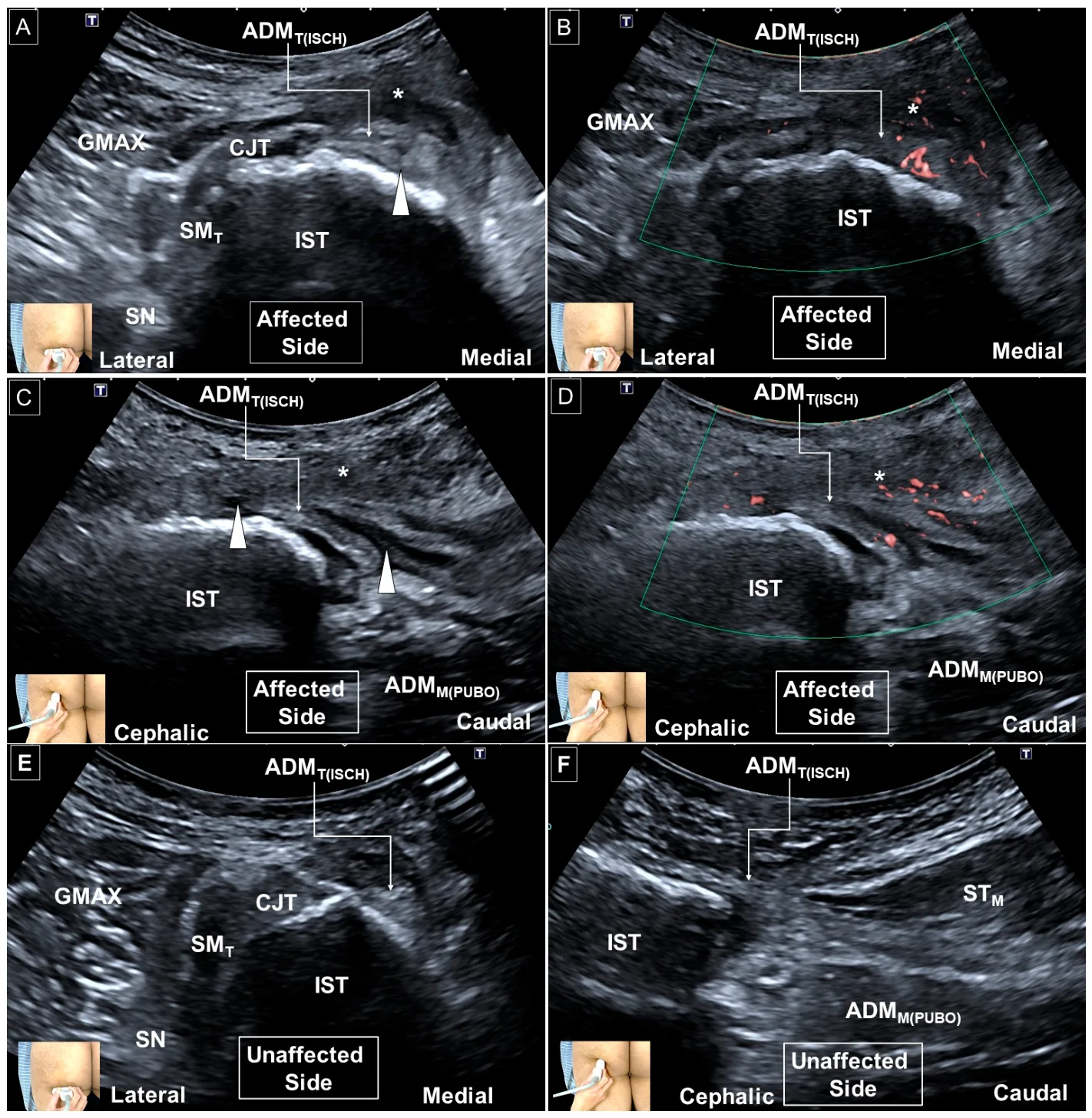
The short-axis grayscale (**A**) and color Doppler (**B**) ultrasonography, together with the long-axis grayscale (**C**) and color Doppler (**D**) image demonstrate a partial tear involving the tendon insertion and musculotendinous junction on the affected side (white arrowhead). Corresponding short-axis (**E**) and long-axis (**F**) ultrasound images of the contralateral (unaffected) side are shown for comparison. Asterisk: ischial tuberosity bursa. CJT, conjoint tendon; GMAX, gluteus maximus; SM_T_, semimembranosus tendon; SN, sciatic nerve; IST, ischial tuberosity; ADM_T(ISCH)_, ischiocondylar portion of the adductor magnus tendon; ADM_M(PUBO)_, pubofemoral portion of the adductor magnus muscle. The ultrasonographic images were obtained from a 63-year-old woman who presented with a 2-month history of left proximal posterior thigh pain. The symptoms had begun after a sprint with rapid acceleration and turning on a running track. She subsequently switched to stationary cycling for exercise; however, she gradually developed marked tenderness beneath the left buttock, which was aggravated by prolonged sitting. The patient reported a sensation of tightness while sitting cross-legged, accompanied by reduced running endurance. Oral nonsteroidal anti-inflammatory drugs provided only limited symptomatic relief, and local heat application paradoxically worsened the sensation of fullness and pain. Inspection revealed no ecchymosis or palpable mass. Physical examination demonstrated focal tenderness just distal and medial to the ischial tuberosity. Pain was exacerbated by resisted hip adduction and hip extension. Muscle strength testing revealed mild weakness during resisted hip extension, while passive hip flexion stretching elicited sharp pain over the proximal posterior thigh. Musculoskeletal ultrasound examination was performed with the patient in the prone position using an Aplio i700 ultrasound system equipped with a curvilinear transducer (10C3, 3.6–9.2 MHz; Canon Medical Systems Corporation, Otawara, Japan). The transducer was initially placed gently over the ischial tuberosity with minimal pressure to obtain a short-axis view. The conjoint tendon, formed by the long head of the biceps femoris and the semitendinosus muscle, was identified originating from the medial and posterior aspects of the ischial tuberosity. Just distal to the conjoint tendon attachment, the proximal origin of the ischiocondylar portion of the adductor magnus was visualized medial to the conjoint tendon. The ischiocondylar portion was then traced distally, and the transducer was pivoted from a proximal-lateral to distal-medial oblique orientation to obtain its long-axis view. The symptomatic and contralateral asymptomatic sides were examined using the same anatomical landmarks and scanning sequence. Dynamic maneuvers were not performed. The examination was performed by an attending physician with more than 15 years of experience in musculoskeletal ultrasonography, using a standardized bilateral scanning protocol to ensure consistent anatomical identification and comparison. Given the examiner’s extensive experience, the sonographic findings were interpreted directly by the examining physician and were not subjected to additional independent assessment by a second specialist. The painful left side on short-axis (**A**,**B**) and long-axis (**C**,**D**) ultrasonography revealed a focal hypoechoic defect with loss and disruption of the normal fibrillar echotexture, suggestive of a partial-thickness tear involving the tendon origin and musculotendinous junction of the ischiocondylar portion of the adductor magnus. These abnormalities were absent on the contralateral asymptomatic side (**E**,**F**). Color Doppler imaging demonstrated adjacent hypervascularity, supporting local inflammatory changes. A hypoechoic ischial bursa was also noted. The focal structural abnormality of the ischiocondylar adductor magnus corresponded to the site of maximal tenderness and was clinically supported by pain during resisted hip adduction and extension. The adjacent hypoechoic ischial bursa and surrounding hypervascular changes were regarded as concomitant findings. However, given the coexistence of these abnormalities, ischial bursitis may also have contributed to the patient’s symptoms, and the relative contribution of the tendon lesion and bursal inflammation cannot be determined from this single case. The patient was advised to reduce prolonged sitting, use an appropriate cushioning device, and modify sporting activities to minimize persistent compression over the affected region. Sprinting, rapid acceleration, and cross-legged yoga postures were temporarily avoided to reduce mechanical stress on the affected tendon. A conservative rehabilitation program consisting of low-level laser therapy and topical nonsteroidal anti-inflammatory medication targeting the symptomatic region was also initiated. Following these multimodal conservative managements, the patient’s symptoms gradually resolved, with no recurrence during more than two months of follow-up. Because activity modification, low-level laser therapy, and topical nonsteroidal anti-inflammatory medication were implemented concurrently, the observed clinical improvement cannot be attributed to any single therapeutic component.

**Figure 2:**
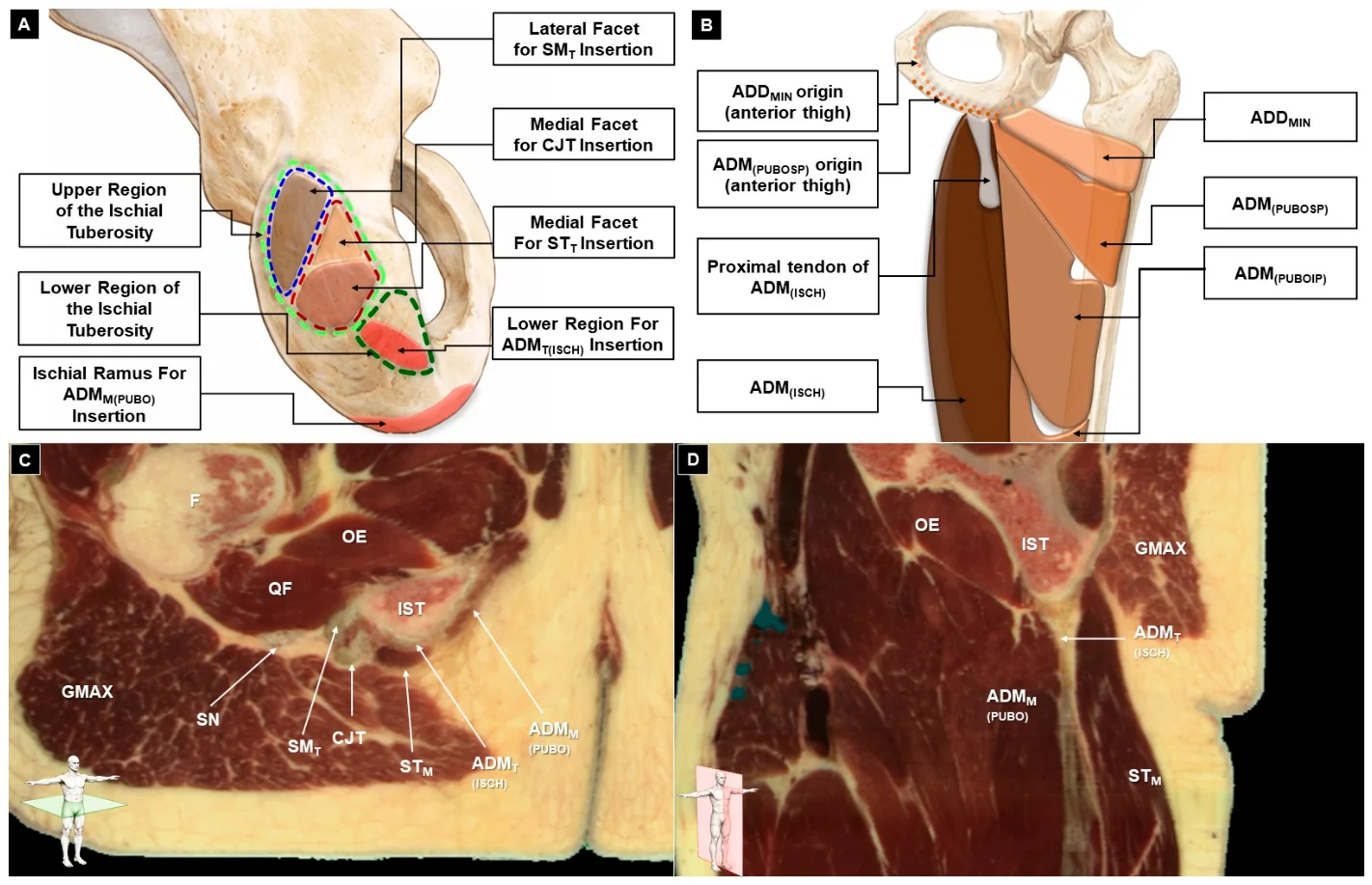
Anatomy of the proximal ischial region and adductor magnus. (**A**) Proximal attachments at the ischial tuberosity, subdivided into upper (light green dashed area), which comprises the lateral (blue dashed area) and medial (red dashed area) facets, and lower (dark green dashed area) regions. (**B**) Posterior view of the ischiocondylar and pubofemoral portions of the adductor magnus. Corresponding cadaveric transverse (**C**) and sagittal (**D**) section images from the Visible Human Project^®^ (used with permission from Touch of Life Technologies Inc.) exemplify the complex regional anatomy. CJT, conjoint tendon; GMAX, gluteus maximus; SM_T_, semimembranosus tendon; ST_M_, semitendinosus muscle; SN, sciatic nerve; IST, ischial tuberosity; ADM_T(ISCH)_, ischiocondylar portion of the adductor magnus tendon; ADM_M(PUBO)_, pubofemoral portion of the adductor magnus muscle; QF, quadratus femoris; F, femur; OE, obturator externus; ADM, adductor magnus; ADD_MIN_, adductor minimus; ADM_M(PUBOSP)_, superior part of pubofemoral portion of the adductor magnus muscle; ADM_M(PUBOIP)_, inferior part of pubofemoral portion of the adductor magnus muscle. The anatomy of the ischial tuberosity is highly complex because multiple tendons share a common origin or insert in close proximity. This intricate anatomical arrangement makes accurate identification of the injured structure challenging and may lead to misdiagnosis or inappropriate treatment. The ischial tuberosity is divided into upper and lower regions [1]. The upper region contains a lateral facet as the attachment for the semimembranosus tendon and a medial facet as the attachment for the conjoint tendon and proximal semitendinosus muscle (**A**,**B**). The lower region serves as the origin of the ischiocondylar portion of the adductor magnus (**A**), while the pubofemoral portion attaches more medially from the ischial ramus (**C**). During ultrasound examination, the transducer can be first placed at conjoint tendon attachment of the ischial tuberosity, with the clear observation of the gluteus maximus, semimembranosus tendon, conjoint tendon and the sciatic nerve. Just distal to the conjoint tendon attachment of upper region, the proximal origin of the ischiocondylar portion of the adductor magnus is seen to arise from the lower region of the ischial tuberosity (**D**). As the largest muscle of the medial thigh compartment, the adductor magnus consists of three anatomically distinct components: the adductor minimus, the pubofemoral portion and the ischiocondylar portion (**B**) [2]. The adductor minimus forms the most superior portion of the muscle, characterized by a distinct fascicular organization. The adductor minimus originates from the anterior surface of the inferior pubic ramus and ischial ramus. It inserts from the lower border of the quadrate tubercle, passing between the gluteal tuberosity and lesser trochanter, and terminating at the lateral lip of the linea aspera. The adductor minimus receives innervation from the posterior branch of the obturator nerve. The pubofemoral portion of the adductor magnus is further divided into superior and inferior parts (**B**) [2]. The superior part inserts along the upper half of the lateral lip of the linea aspera, medial to the insertion of the gluteus maximus. The inferior part inserts along the lower half of the medial lip of the linea aspera and the medial supracondylar line. The pubofemoral portion originates from the inferior pubic ramus and the ischial ramus [3]. Compared with the ischiocondylar portion, its fibers are more anteriorly located and horizontally oriented. During ultrasound examination for the ischiocondylar portion, the transducer is initially positioned over the conjoint tendon attachment and subsequently pivoted from a proximal-lateral to distal-medial oblique orientation, rather than maintaining a true sagittal plane, to visualize the long axis of the ischiocondylar adductor magnus tendon. In this view, the pubofemoral portion of the adductor magnus is visualized anterior to the ischiocondylar portion. The principal differential diagnoses for pain arising from this region include proximal hamstring tendinopathy or tear and ischial bursitis. On ultrasonography, proximal hamstring pathology is centered on the semimembranosus or conjoint tendon origins at the upper region of the ischial tuberosity and may manifest as tendon thickening, hypoechogenicity, loss of the normal fibrillar echotexture, or focal fiber discontinuity. In contrast, abnormalities of the ischiocondylar adductor magnus are located more distally and medially and can be distinguished by tracing the affected fibers along the characteristic oblique course of the muscle and tendon. Ischial bursitis is characterized predominantly by distention of the bursa with hypoechoic or anechoic fluid adjacent to the ischial tuberosity, with possible synovial thickening and surrounding hypervascularity. Careful localization of the abnormality, correlation with the site of maximal tenderness, and comparison with the contralateral side may therefore facilitate differentiation among these closely related causes of ischial pain. Both the superior and inferior parts of pubofemoral portion receive innervation from the posterior branch of the obturator nerve. In contrast, the ischiocondylar portion arises from the ischial tuberosity and exhibits a unipennate muscle architecture. Distally, its fibers converge into a thick, cord-like tendon that inserts onto the adductor tubercle of the medial femoral epicondyle, with a subset of fibers extending to the medial supracondylar line [3]. This portion is innervated by the tibial division of the sciatic nerve. All three portions of the adductor magnus receive their primary arterial supply from the profunda femoris artery, with additional vascular contributions from the medial circumflex femoral and popliteal arteries. The adductor magnus occupies both the medial and posterior compartments of the thigh and plays an important role in hip adduction and extension [4]. Several factors increase the risk of muscle injuries, including muscle imbalances, limited flexibility, inadequate warm-up and prior injuries. Acute injuries of the adductor magnus are common in activities involving kicking, excessive eccentric muscle contraction, or rapid changes in direction, such as soccer and skiing, and most frequently occur at the musculotendinous junction [5,6]. Although adductor magnus injuries are considered uncommon, they may be diagnostically challenging because of their deep location and proximity to adjacent structures. In particular, the ischiocondylar portion, which originates directly from the ischial tuberosity, is difficult to differentiate on magnetic resonance imaging because its pathology is often obscured by concomitant ischial bursitis or adjacent proximal hamstring injuries. High-resolution ultrasonography has emerged as a valuable imaging modality for evaluating the proximal hamstring and adductor complex, providing real-time, dynamic assessment of muscle and tendon morphology [7]. Its ability to delineate subtle structural abnormalities and identify musculotendinous abnormalities may facilitate the recognition of lesions in this anatomically complex region and assist in image-guided management. MRI was not performed in this case; therefore, independent cross-sectional imaging confirmation of the suspected partial tear was unavailable. Although the ultrasonographic abnormalities corresponded to the symptomatic region and differed from those on the contralateral side, the diagnosis should be interpreted in the context of this limitation.

## Data Availability

The original contributions presented in the study are included in the article; further inquiries can be directed to the corresponding authors.
